# Changes in Gambling Behavior during the COVID-19 Pandemic—A Web Survey Study in Sweden

**DOI:** 10.3390/ijerph17114013

**Published:** 2020-06-05

**Authors:** Anders Håkansson

**Affiliations:** 1 Department of Clinical Sciences Lund, Psychiatry, Faculty of Medicine, Lund University, S-22100 Lund, Sweden; anders_c.hakansson@med.lu.se; 2 Region Skåne, Gambling Disorder Unit, S-20502 Malmö, Sweden

**Keywords:** COVID-19, gambling disorder, problem gambling, behavioral addiction, pandemic, crisis

## Abstract

The COVID-19 pandemic has dramatically changed everyday life, and policy makers have raised concerns about possible changes in gambling patterns during the pandemic. This study aimed to examine whether self-reported gambling has increased during the pandemic, and to examine potential correlates of such a change. This general population survey study in Sweden collected self-report data from 2016 web survey members (51 percent men, nine percent moderate-risk/problem gamblers). Correlates of increased gambling and increased gambling specifically due to COVID-19-related cancellation of sports were calculated. Four percent reported an overall gambling increase during the pandemic. The proportion of individuals reporting an increase, compared to individuals reporting a decrease, was markedly higher for online casinos (0.62), online horse betting (0.76) and online lotteries (0.73), and lower for sports betting (0.11). Overall, gambling increases were independently associated with gambling problems and increased alcohol consumption. In the sub-group, where there was an increase in specific gambling types in response to cancelled sports betting events, rates of gambling problems were high. In conclusion, only a minority report increased gambling in response to the pandemic, but this group has markedly higher gambling problems and changes in alcohol consumption, and may represent a sub-group with a particularly high vulnerability. This calls for preventive action in people with higher gambling risks in response to the pandemic.

## 1. Introduction

The ongoing COVID-19 crisis has had a broad and deep impact on people’s lives globally, and in addition to the physical harm from the pandemic, it is believed to have a high degree of impact on the mental health of the population [1,2]. Among the potential consequences of the pandemic and its impact on everyday life, it has been suggested that addictive behaviors may be enhanced [3]; this may, for example, include a risk of increased problematic online gaming [4].

Based on the impact described above from the COVID-19 pandemic, it is possible that gambling behavior could also be altered because of the pandemic [5]. Gambling is a well-established potentially addictive behavior, and gambling disorder is a diagnostic entity along with alcohol and drug use disorders in the modern psychiatric diagnostic framework [6], and problem gambling, with or without the diagnostic level, is reported to affect around one percent to around five percent of the world-wide population [7]. While the current situation is obviously one that has never been seen before, previous larger national financial crises have been described to affect gambling behavior. After the deep financial crisis in Greece, starting in 2008, it was said that having started gambling during the financial crisis was a risk factor of later developing gambling problems [8]. Findings from the financial crisis of Iceland, starting with the bank crash in 2008, demonstrate that a crisis may increase specific types of gambling behavior. While it could be seen that lottery gambling may increase during financial hardship [9,10], it was not clearly demonstrated that the financial crisis had an impact on problem gambling rates in the population [10]. Previous findings also describe a mixed picture; lottery gambling may not be sensitive to income decreases during recession, while casino gambling tends to diminish in difficult times [11].

The COVID-19 crisis presents several potential challenges which could theoretically affect gambling behavior in an unforeseen manner. Over and above the financial crisis and uncertainty around the economic future, the present crisis leads to dramatic changes in the amount of time spent at home, and likely increases time spent online [4]. Likewise, the gambling market has been dramatically altered in only a few weeks, as major sports events worldwide have been cancelled or postponed. The decrease in opportunities for sports-related gambling may theoretically either decrease gambling in sports bettors, or increase some gamblers’ involvement in other gambling types which they may or may not otherwise use. The fear of sports bettors transferring to potentially more hazardous gambling has been described by policy makers in the present setting, pointing particularly towards online casinos [12] as a potentially more hazardous gambling opportunity [13]. The overall concerns about an altered gambling behavior during the crisis have led several governments to take action through different measures, such as a limitation on gambling advertisements in Spain [14], deposit limits in Belgium [15], and a total ban in Latvia [16].

In light of the novelty of the present situation, structured data from the general population is lacking and needed in order to deepen our understanding of how gambling behavior may be changing due to the current pandemic. Moreover, policy decisions currently taken by different governments are diverse and need to be accompanied by systematic research data. Market data report, for instance, a substantial increase in horse race betting in the present setting [17], although it is difficult to derive this from how it affects the general population’s actual gambling behavior, and whether this affects particular sub-groups more than others. For this reason, the present study, a general population web survey, was carried out in midst of the COVID-19 pandemic in Sweden. The study aimed to address how self-reported gambling behavior may have changed overall and for specific gambling types, and how potential changes in gambling may be associated with specific risk factors or with lifestyle changes such as increased time at home or increased alcohol consumption. Given the dramatic decrease in sports-related gambling, it specifically aimed to address how other gambling types may have changed in response to this.

## 2. Methods

The present study is a cross-sectional web survey study addressing a general population sample enrolled from the web panel of a market survey company in Sweden. The study was reviewed by the national Swedish Ethical Review Authority, which decided the project did not require formal ethical approval (as it does not include data that can be linked to identified individuals), and expressed that it had no ethical concerns with respect to the present project (file number 2020-01856).

### 2.1. Setting

The present general population-based survey study was carried out in Sweden in response to the potential influence on gambling-related behaviors from the COVID-19 pandemic. In the past decade, gambling in Sweden has moved towards a higher degree of online gambling and problem gambling in treatment-seeking patients and is primarily reported to occur online, with online casino gambling being the most common type of problematic gambling reported [18]. In recent years, gambling advertising has expanded considerably, and gambling advertising is strongly predominated by online-based gambling and most commonly online casino gambling. Besides online casino gambling, sports betting is commonly exposed in gambling advertisements [19] and reported by treatment-seeking patients, primarily in men [18]. In the general population, around 60 percent reported gambling in the past year, and among gamblers, lotteries (75 percent), chance-based number games (50 percent), horse racing (38 percent), and sports betting (21 percent) are the most common gambling types, whereas bingo (11 percent), casino gambling (5 percent) and poker (4 percent) are less frequent in the whole population. Among gamblers, 48 percent reported gambling in gambling shops, 29 percent on mobile telephones, 19 percent on computers, and nine percent on a tablet; in contrast, only 4 percent reported gambling on horse race tracks and 1 percent reported gambling in a physical casino. In particular, gambling on a mobile telephone has increased clearly in recent years [20]. Past-year problem gambling has been reported to be comparable to or towards the higher end of problem gambling rates reported in other European settings, with problem gambling (measured with thee PGSI, Problem Gambling Severity Index) reported to be around 0.3 percent, and SOGS-R (South Oaks Gambling Screen-revised) past-year problem and pathological gambling at a total of 2.2 percent of the population [7]. From previously being an officially monopoly-based system with many unlicensed overseas online gambling companies [13,19], since 1 January 2019, the Swedish gambling market includes a large number of licensed and thereby regulated gambling operators. Furthermore, in line with previous experience of self-exclusion from gambling [21], this system also includes a novel national self-exclusion service (called *Spelpaus*), which covers all licensed gambling operators (such that a person who chooses to self-exclude can do so from all operators through one national governmental authority).

The COVID-19 crisis has led to significant constraints on society world-wide. Although adopting a less restraining policy, Swedish authorities early in the course of the pandemic prohibited public gatherings of more than 500 people, and thereafter with a change to 50 people, thereby making sports events virtually impossible [22]. Like in other countries, this has significantly changed the gambling market. As a symptom of the same development, gambling was reported to attract international operators in surprisingly low-tier training games [23]. Concerns over a possible negative impact on gambling behaviors in the population has led to government initiatives aiming for a potentially more restricted gambling market policy [12], following other political policy initiatives internationally [14,15,16].

### 2.2. Study Procedures

Web panel members were invited by a web link which provided information about the study, with the aim to include participants until 2000 complete responses were reached and included an even distribution of women and men, with an acceptable distribution across age groups. The study was carried out during a period of ten days, from 24 April, through 3 May 2020. The questionnaire data was directed immediately to the companies Patient Information Broker (PIB, Landskrona, Sweden) and I-Mind Consulting (Lund, Sweden), who are responsible of the technical set-up of the survey, and response data were therefore not sent to the web panel operator. The researcher, as well as the companies PIB and I-Mind Consulting collecting the data, were unaware of the identities of the people enrolled in the web panel.

### 2.3. Participants

Study participants were members of the web panel of a market survey company, Userneeds (userneeds.com, Copenhagen, Denmark), whose web panel members regularly receive different types of surveys, typically market surveys. Members of the web panel received a link to a survey, and voluntarily accepted to fill out the survey. Participation was rewarded with credits through the company’s own internal credit system, with a value corresponding to around 1.50 Euros; the present type of study recruitment, from the same company, has been carried out by the research group in several recent publications in the present field of research [24,25,26].

Participants were all over 18 years of age, and were able to open the survey only after receiving the study information and after actively consenting to study participation. The survey did not include any information that could directly or indirectly identify an individual. Moreover, IP-addresses or the geographical location of respondents could not be detected by the researcher; however, coded information of IP-addresses was used in order to identify potential duplicate answers. In such cases, if the first answer was a complete survey answer, that data was included, whereas duplicates were excluded. Nineteen individuals who had suspected duplicates in the system and for whom the first response was incomplete were not considered further in the study and were not included in the study sample addressed below.

### 2.4. Measures

Basic socio-demographic variables included gender, age (in broad age groups), monthly income, living conditions, and occupation (Table 1). In the analyses below, occupation was divided into categories describing those without a regular occupational status, i.e., on sick-leave, job-seeking, or short-term unemployed (the latter being added and worded in the way these short-term changes in the labor market are referred to during the COVID-19 crisis). The questionnaire also collected dichotomous data on whether a respondent had or had not gambled on each specific gambling platform during the past year (online casino, land-based casino, online horse betting, land-based horse betting, online sports betting, land-based sports betting in betting shops, online poker, land-based electronic gambling machines, and online bingo). Thereafter, the questionnaire opened a section starting with brief wording on the specific situation that COVID-19 leads to, such as changes in working life or everyday activities, with questions about changes in the individual’s behavior during the COVID-19 pandemic (“since these changes in Sweden started”); whether she/he, during this period, had spent more or less time at home (much more, slightly more, unchanged or less time at home); and had consumed more or less alcohol (more alcohol than prior to the pandemic, less alcohol than during the pandemic, unchanged, or “don’t drink at all, neither now nor before”). Thereafter, the corresponding question was asked for gambling, and it was specified that gambling refers to gambling for money and that this may include gambling on sports, casino, bingo, card games, or lotteries, either online or on land-based platforms. Options included “gamble more during COVID-19 than before”, “gamble less during COVID-19 than before”, “no, completely unchanged”, and “I don’t gamble, neither now nor before”. Later detailed questions were asked in the same way but for each type of gambling: online casino; online sports betting; land-based sports betting (in betting shops); online horse gambling; land-based horse gambling; online lotteries; land-based lotteries; and land-based electronic gambling machines, respectively. Furthermore, questions were asked about total gambling losses from the past 30 days, and thereafter total gambling losses during a “typical 30-day period”, specifying that losses refer to money actually lost (in order to clearly separate the wording from gambling deposits). Another gambling-related question that was asked was whether the respondent had ever self-excluded from gambling through the national self-exclusion system (*Spelpaus*; “yes”, “no”, or “prefer not to say”).

One specific question asked whether the respondents’ gambling has changed as many sports events have disappeared during the crisis: “bet more on other types of sport games than before”; “gamble more on horses”; “gamble more on online casino”; “gamble more on other games”; “gamble less overall”; or “doesn’t affect me at all—I don’t gamble on sports”.

In addition to the questions above, the level of potential gambling problems was measured with the 9-item Problem Gambling Severity Index (PGSI [27]), where each of the statements addresses the past 12-month period, with options including “never”, “sometimes”, “most of the time”, and “almost always”. Psychological distress was measured using the Kessler-6 (K-6) scale (Furukawa et al., 2003 [28]), with six questions addressing depressive and anxiety-related symptoms during a time frame of the past six months, with options ranging from “not at all” to “all the time”, also including a “cannot answer/prefer not to say” response. For this scale, the scores (0–4 for each question) were summed, and a total score of five or more was classified as at least moderate psychological distress. In 54 cases where at least one of the six K-6 items were missing (“cannot answer/prefer not to say”), from 24 of these, it could be concluded from the available answers whether or not an individual reached the cut-off of 5 for the available responses, or whether they had a total score of 0 from five responses, thus making the full score below 5 regardless of the hypothetical value of the single missing response. The remaining 30 cases, where the level above/below the cut-off could not be established, were excluded from this calculation. In addition to the reporting of moderate psychological distress, for descriptive purposes, distribution of full Kessler-6 scores were also reported.

### 2.5. Statistical Methods

The analyses on whether respondents increased their gambling (separated from all other options) were calculated for the overall gambling question, as well as for each of the specific gambling types addressed with respect to their potential change during COVID-19. The overall gambling increase item was analyzed with respect to several potential risk factors (see Table 2), and the items significantly associated with a gambling increase in these chi-squared bivariate analyses were further entered in a logistic regression; due to the risk associated with multiple analyses, as 10 items were tested for potential associations, the significance level for inclusion in the logistic regression was set at *p* = 0.005 (due to a Bonferroni correction, 0.05/10). For measured increases in each of the gambling types, due to a more limited number of respondents reporting the outcome and for a direct comparison across gambling types, logistic regressions were carried out including the items male gender (vs female/prefer not to say), age groups, whether or not a respondent had spent more time at home, and the level of problem gambling severity (no risk, low risk, moderate risk, or problem gambling). For the question addressing how gambling had changed in response to the cancellation of sports events, where more than one answer could be chosen, each possible response was analyzed with respect to univariate associations and logistic regression, all including the same four variables as potential correlates, chosen in order to keep the number of correlates limited to a few key variables given the low number reporting each outcome (age, gender, time at home, and gambling severity). The final analyses only included individuals who had opted for another response to the overall gambling question than the option “I don’t gamble, neither now nor before”, in order to calculate statistical correlates of change only among individuals with a possible gambling involvement. The remaining analyses were mainly descriptive, and included descriptive percentages as well as the description of the ratio between the number of respondents reporting a gambling increase and the number reporting a decrease (including for each gambling type and for alcohol consumption as a comparison). All calculations were carried out in SPSS version 25.0 (SPSS Inc., Chicago, IL, USA).

## 3. Results

Characteristics of the included sample are displayed in Table 1. A total of 84 percent of respondents were classified to have no-risk gambling, 7 percent (n = 145) reported low-risk gambling, 4 percent (n = 76) moderate-risk gambling, and 5 percent (n = 98) problem gambling. Thus, a total of 9 percent (n = 174) were either moderate-risk or problem gamblers. The median total PGSI score of the sample was 0 (inter-quartile range 0–0, 90th percentile 1, range 0–27). Three percent (n = 70) had self-excluded from gambling in the national *Spelpaus* self-exclusion system, whereas 95 percent had not, and 2 percent did not wish to respond (one missing case for this variable).

### 3.1. Behavior Change during COVID-19

Forty-five percent (n = 912) reported spending much more time at home during the COVID-19 crisis, 34 percent (n = 679) spent slightly more time at home, 20 percent (n = 407) reported no difference, and 1 percent (n = 18) reported spending less time at home. Eight percent (n = 161) reported drinking more alcohol during the COVID-19 crisis, 65 percent (n = 1312) reported no difference, 10 percent (n = 210) reported drinking less, and 17 percent (n = 333) reported drinking no alcohol, neither now nor prior to the crisis.

### 3.2. Overall Changes in Gambling Patterns during COVID-19

Four percent (n = 74) reported gambling more during the COVID-19 crisis, 51 percent (n = 1027) reported no difference, 7 percent (n = 145) reported gambling less, and 38 percent (n = 770) reported no gambling, neither now nor prior to the crisis. For nine percent (n = 185), the category of monthly gambling loss for the past 30-day period was higher than the category chosen for the question on a typical 30-day period. The proportion of individuals reporting an increase as a proportion of those reporting a decrease, was 0.51 for gambling in general. For comparison, the corresponding figure for alcohol consumption was 0.77.

Among those who reported any gambling (n = 1246, i.e., except respondents who reported that they do not gamble at all, neither now nor prior to the crisis), 59 percent of the sample were men, 77 percent reported being more at home, and the percentages of moderate-risk and problem gambling were six and seven percent, respectively, a total of 13 percent. In this sub-group, the 74 individuals reporting increased gambling represented six percent.

### 3.3. Correlates of Increased Gambling—Gambling Overall

In the sub-group of all individuals, other than those reporting no gambling now or before (n = 1246), in univariate chi-square analyses, gambling more was significantly associated with a higher gambling problem severity (*p* < 0.001), younger age (*p* < 0.001), more time at home (*p* = 0.01), higher alcohol consumption (*p* < 0.001), psychological distress (*p* < 0.001), and a history of self-exclusion (*p* < 0.001), whereas it was unrelated to living alone without children (*p* = 0.90), monthly income (*p* = 0.64), gender (*p* = 0.87), and occupation (*p* = 0.12). When entering all variables with a *p* < 0.005 into a logistic regression, increased gambling remained associated with higher problem gambling severity, and with increased alcohol consumption during the pandemic. The same list of independent associations was seen when analyzing the full sample (Table 3).

### 3.4. Correlates of Increased Gambling—Separate Gambling Types

For each specific type of gambling, the percentage reporting an increase (among those not excluding gambling for that specific type), and the ratios of individuals reporting an increase to those reporting a decrease, are shown in Table 4. Each of the gambling types were analyzed including time at home, gender, age, and gambling severity as potential risk factors in logistic regression analyses. An increase in online casino gambling was associated with gambling severity (*p* < 0.001) and younger age (*p* = 0.05, significant, rounded off to 0.05). Increased online sports gambling was associated with gambling severity (*p* < 0.001). Increased land-based sports gambling was associated with gambling severity (*p* < 0.001) and younger age (*p* < 0.01). Increased online horse betting was associated with gambling severity (*p* < 0.001) and older age (*p* = 0.01). Increased land-based horse betting was associated with gambling severity (*p* < 0.001). Increased online lotteries were associated with gambling severity (*p* < 0.001), and increased land-based lotteries were associated with gambling severity (*p* < 0.001), female gender (*p* = 0.02) and with spending more time at home (*p* = 0.02). Increased machine gambling was associated with gambling severity (*p* < 0.001, in the latter analysis, spending time at home could not be included, due to zero individuals in one of the groups).

### 3.5. Changes in Gambling Patterns in Response to Decreased Sports Betting

In the sub-sample of individuals who reported gambling, i.e., other than those who reported no gambling now or before (n = 1246), in response to the decreased market of sports betting, two percent (n = 28) reported gambling more on other sports games, six percent (n = 78) reported more horse betting, four percent (n = 44) reported more online casino gambling, five percent (n = 65) reported more of other games, 19 percent (n = 232) reported gambling less, and 69 percent (n = 857) reported that they do not gamble on sports and are therefore unaffected by its decrease.

In unadjusted analyses, gambling more on other sports games was significantly associated with being male (*p* = 0.03), gambling severity level (*p* < 0.001, 71 percent were problem gamblers and a total of 82 percent were moderate-risk or problem gamblers), and younger age (*p* < 0.001), but not with increased time at home (*p* = 0.12). Increasing horse betting was associated with male gender (*p* = 0.03), gambling severity level (*p* < 0.001, 31 percent were problem gamblers and 49 percent were either moderate-risk or problem gamblers), and with time at home (*p* = 0.03), but not with age (*p* = 0.10). Gambling more in online casinos was associated with male gender (*p* = 0.01), gambling problem severity (*p* < 0.001, 64 percent problem gamblers and a total of 89 percent were moderate-risk or problem gamblers), and younger age (*p* < 0.001), but not with time at home (*p* = 0.70). Gambling more on other games was associated with male gender (*p* < 0.001), problem gambling severity (*p* < 0.001, 43 percent problem gamblers and a total of 52 percent were moderate-risk or problem gamblers), and younger age (*p* < 0.001), but not with spending more time at home (*p* = 0.24).

An endorsement of the response that one gambles less in total in response to reduced sports gambling was associated with male gender (*p* < 0.001), higher gambling problem severity (*p* = 0.03, seven percent were problem gamblers and a total of 15 percent were either moderate-risk or problem gamblers), and younger age (*p* = 0.01); this was also marginally associated with more time at home (*p* = 0.06). Those that reported not being sports bettors were more likely to be women (*p* < 0.001), to have a lower degree of problem severity (*p* < 0.001, two percent problem gamblers and a total of four percent were moderate-risk or problem gamblers), a higher age (*p* < 0.001) and were more likely to not spend more time at home (*p* < 0.001).

In logistic regression including the same four potential risk factors, betting more on other sports was associated with problem gambling severity (*p* < 0.001), more horse betting was associated with problem gambling severity (*p* < 0.001) and older age (*p* = 0.01), gambling more in online casinos was associated with problem gambling severity (*p* < 0.001), gambling more on other games was associated with problem gambling severity (*p* < 0.001) and with male gender (*p* = 0.01), and gambling less was associated with male gender (*p* < 0.001) and younger age (*p* = 0.03 and unrelated to problem gambling), and a report of no sports gambling of any sort was associated with lower gambling severity (*p* < 0.001), female gender (p < 0.001), older age (p < 0.05), and with not spending more time at home (*p* < 0.01).

## 4. Discussion

The present study addressed a question hitherto not described in empirical research data, i.e., whether and how gambling habits may have changed in response to the current COVID-19 pandemic. While the present general population web survey cannot describe causality of associations, it has demonstrated several relevant findings. While the majority of respondents did not report altered gambling habits, and the proportion reporting an increase was smaller than the proportion reporting a decrease, a significant minority of respondents still reported increasing their gambling, and a consistent finding—both for overall gambling and for specific gambling types—was that this sub-group had markedly higher gambling problems. Moreover, those increasing their gambling had an increased alcohol use during the pandemic, even when controlling for several other potential risk factors. Another important finding was that the minority of respondents reporting an increase of other gambling in response to the rapid shortfall of sports events, had very high rates of gambling problems. One overall impression is that while the pandemic does not demonstrate changes in the population as a whole, for a sub-group of individuals with high vulnerability, increased gambling may be a real problem and may need to be targeted with interventions.

### 4.1. Gambling Severity among Those Reporting an Increase

The study demonstrated that a non-negligible percentage of respondents reported an increase in gambling behavior during the COVID-19 crisis. In the present study, it is not possible to conclude how the changes (increases and decreases) in gambling behavior correspond to natural fluctuations regularly happening in the general population. However, importantly, the group reporting increased gambling behaviors differed from other respondents; consequently, across different analyses and measures in the study, increased gambling was independently and clearly associated with the problem of gambling severity. Among those reporting increased gambling, more than half of respondents were moderate-risk or problem gamblers, and a history of self-exclusion was reported by a surprisingly high 28-percent proportion of that group.

The changes in relation to the dramatically altered sports gambling market are of relevance to the current situation, and may inform stakeholders in the gambling area on relevant measures in the current situation. Here, again, the minority reporting a switch to other gambling had a clear picture of problematic gambling involvement. While some sports gambling still may occur even during the most extensive COVID-19-related restraints, such as games in leagues that very rarely appear in the media or unexpected betting on training or low-tier soccer games [14,23], within the minority who reported increasing “other sports betting”, more than four out of five were categorized as moderate-risk or problem gamblers. Likewise, a switch to online casinos was reported by a group where around nine out of ten were moderate-risk or problem gamblers. For the group reporting a switch to horse betting, the percentage with gambling problems was smaller, but nevertheless, around half were at least moderate-risk gamblers. Thus, this leads to a possible conclusion that when the world of sports is nearly entirely cancelled, those who still seek other gambling involvement may be a group that are important to address with preventive measures.

Again, the association of gambling problems with a reported switch from sports betting to online casinos confirms the high addictive potential of online casino gambling. In recent research from the present setting, problem gambling and indebtedness were markedly higher if online casinos were part of the past-month gambling pattern [13], online casinos are the leading gambling platform cited by treatment-seeking patients [18]. One factor possibly contributing to this is the high exposure to online casinos specifically in television advertisements [19]. It may be possible to assume that time at home during the COVID-19 pandemic could potentially affect online gambling especially, rather than other types [29].

The present findings cannot demonstrate whether full gambling expenditures or the gambling involvement of the general population has increased, except that there is a slight tendency towards an increase in self-reported deposits during the past month compared to a “typical” month. However, although the present research question is obviously new and findings have to be interpreted with caution, this study gives support to the fear that a sub-group of highly involved sports bettors may transfer to other gambling platforms such as online casinos, and that this may provide preliminary evidence to advise gamblers not to replace their gambling with other gambling types when their preferred type of gambling is cancelled.

### 4.2. Role of Online Gambling in Potential Behavior Change

Certain types of gambling were more likely than others to have a higher percentage of respondents reporting an increase, compared to those reporting the opposite. Except for online sports betting, which is likely decrease due to the rapid cancellation of most sports events worldwide, online gambling platforms generally represented a higher proportion of the reported increase in gambling compared to those reporting a decrease. Although these self-reported figures must be interpreted with caution, they lend some support to the notion that replacing one gambling type with another during the pandemic may be of greater relevance for some platforms than for others. Clearly, land-based horse betting may not serve as a viable option for a large majority of sports bettors when sport is cancelled, whereas online casinos and online lotteries, as well as online horse betting, are easily accessible alternative options. Online gambling in general may be associated with a higher risk of addictive behavior and harm [30,31]. Moreover, in literature describing reasons for gambling specifically online, rather than in land-based venues, online gamblers have cited a wide range of reasons for doing so; the increased availability and the comfort of using online gambling services are factors which remain unchanged or have even increased during the pandemic. Likewise, a significant minority of online gamblers cite boredom and other motivators reflecting a negative feedback pattern [29], and it cannot be excluded that such triggers for online gambling account for part of the increase in some gambling types here.

Based on previous literature, it is reasonable to believe that when gambling is used as a response to negative emotions, it is more likely to be triggered by a desire for chance-based games rather than skills-based games [32]. Thus, if the emotional consequences of the COVID-19 pandemic are assumed to influence gambling habits, which is beyond the scope of the present study to conclude, this would be in line with a possible increase in online casino gambling. However, this would rather be the opposite to the findings regarding horse betting. It can be assumed that different mechanisms may come into play for individuals changing their gambling habits in response to the pandemic; the sub-group that reported increasing gambling may reflect an effect of social isolation or emotional concerns, or may reflect the decrease in sports-related betting during COVID-19-related constraints, with horse bettors likely to represent more of the latter.

### 4.3. Gender Aspects on Changed Gambling Habits

In the present study, no association was seen between a self-reported gambling increase and gender. Traditionally, males are more likely than females to develop gambling problems in the general population [33,34], and so far, the male predominance appears to translate into the clinical setting [18,31,35]. However, with time, gambling has been described to become more and more acceptable in women, such that gender differences could potentially start to decrease [36,37]. Possibly as a part of this process, and possibly due to a transfer of land-based gambling into the online setting and therefore away from its traditional arenas, female problem gambling has increased in the present setting in recent years; in a recent public health authority survey, an increase in problem gambling was seen mainly in women [38]. Likewise, in the present setting, recent data demonstrate that the female-to-male ratio may be high in the sub-group of people who have a significant online gambling involvement [13]. Based on this, it would be possible to assume that a risk situation which would theoretically increase online gambling would increase the proportion of women. Meanwhile, particular gender effects could be assumed during the COVID-19 crisis due to the decreased sports betting market, where many participants are known to be men [18,39,40,41]. While the present study could not conclude an overall effect of gender on increased gambling, more research in this area may be needed.

### 4.4. Alcohol Use, Psychological Distress, and Time at Home

In the present study, the group reporting increased gambling had higher rates of psychological distress. While this is not surprising, no association remained when controlling for alcohol consumption and gambling severity. Despite this, it further adds to the impression that individuals increasing their gambling during the crisis represent a vulnerable sub-group of the population. Problem gambling is known to be associated with mental health problems, and as shown previously, the direction of this association cannot be suspected only from the present kind of cross-sectional study data [42].

One of the clearest findings of the study has been that a self-reported increase in alcohol consumption during the pandemic is associated with a self-reported increase in gambling overall, even when controlling for other potential correlates of increased gambling. The possible link between alcohol consumption and gambling has received considerable research attention. However, whether or not a concurrent alcohol consumption increases the risk of gambling has been studied with somewhat mixed results, and all results that has been provided so far are not necessarily applicable to a setting with a high prevalence of online gambling. In land-based casino gamblers, alcohol consumption has not convincingly been shown to increase concurrent gambling [43], and in US past-year gamblers, it has been suggested that the positive link between alcohol use and gambling may not be clear in subclinical drinkers, but is clearer among patients with an alcohol use disorder [44]. In a recent study into online sports bettors in Australia, alcohol use and drug use were associated with placing larger bets [45]. Thus, based on the latter finding, it cannot be excluded that the association between alcohol increases and gambling increases in the present study is based on a direct effect of drinking patterns—or possibly an association in the opposite direction, such that increased gambling could increase alcohol consumption. The direction and causality of this association cannot be established. In addition, the association between spending more time at home and increased gambling was weaker than for alcohol, which remained the only significant correlate aside from gambling severity. Thus, it is also possible that the alcohol variable here represents a proxy of a lifestyle change in general, representing both increased drinking, spending more time at home, and possibly other behaviors.

It is known that the reasons for gambling online may be very diverse, but they often include the ease and access of online gambling options [29]. Thus, it remains possible that the increase in gambling is explained by changes in the everyday lives of a sub-group of the population, or that it is reflected by general effects of the financial crisis in the nation, or changes in the gambling market. While this is hitherto inconclusive, what supports an effect from being at home and changing everyday routines is that some gambling types have a different increase/decrease than others; the increase/decrease ratio for online horse betting was higher than for land-based horse betting, and higher for online lotteries than for land-based lotteries, and generally, increase/decrease ratios for land-based gambling types were low. These changes likely reflect changes in the community from COVID-19-related constraints; occasions for land-based activities are overall more limited than those happening online. More research will be needed in order to understand the full picture of variables associated with COVID-19-related changes in gambling patterns, including the relationship between gambling and alcohol behavior.

## 5. Strengths and Limitations

The present study has strengths and limitations. First, it is—to the best of the author’s knowledge—the first study examining the short-term changes in gambling patterns during the COVID-19 crisis, and therefore already provides data during the ongoing crisis that can advise stakeholders and provide a base for further research. However, obvious limitations of the study relate to the fact that the study is an anonymous web survey, with limitations relating to an inability to ask many questions and to collect more in-depth data. Moreover, the temporality between different variables cannot be established in the present kind of survey-based cross-sectional data collection; most importantly, regarding the association between higher gambling severity (PGSI data) and increases in gambling patterns, it cannot be established whether the endorsement of PGSI items is due to the current COVID-19-related increases specifically, or whether a pre-existing PGSI-measured gambling problem subsequently predicted such a response to the pandemic. Likewise, in the present paper, data on past-year use of each type of gambling was included for descriptive purposes, but were not entered into the logistic regression analyses, the latter in order to reduce the number of correlates used, and because of the uncertainty of whether the reporting of one gambling type would represent the baseline gambling pattern or the new, recent gambling pattern. Thus, further research may need to have a longitudinal study design, and may expand the possibilities to study the pre- and post-prevalence of specific gambling types. The present study results rely on self-reported data, which is a strength to the extent that it addresses a more complete picture than one that can be derived from the technical deposit data of each separate gambling operator, but includes an obvious limitation with respect to the detailed measure of what is perceived by an individual to be an increase, a decrease, or a lack of change. Likewise, the estimation of monthly gambling losses was based on self-reported information and the conclusions from the pre-post-comparison are therefore limited, although a substantial percentage of respondents actually endorsed a higher level of loss during the past 30-day period than in a typical 30-day period. Here, objective data such as consumer credit data or bank account data would have been of significant value. Furthermore, the limited format of the present survey limited the amount of variables assessed; for example, immigrant background, a variable known to have an influence on gambling and problem gambling [24,46], was not assessed, presenting another limitation to the present work.

The present study was carried out as a web survey addressing the general population, specifically members of a web panel from a market survey company. Related to this, we can conclude that problem and/or moderate-risk gambling was markedly higher than what has been reported in general population studies from the same setting [47]. Thus, it is very likely that a web survey addressing gambling-related issues may attract a sample of respondents with a higher involvement in—or interest in—gambling, which also has been seen in previous studies from our group using a similar methodology and in samples recruited in the same way [24,25,26].

## 6. Conclusions

The present general population web survey study, carried out after only a number of weeks with COVID-19-related constraints, was able to demonstrate self-reported changes in gambling behavior in response to the pandemic. Although the number of individuals reporting a gambling increase was smaller than the number reporting a decrease, and although the large majority reported no change, it can be concluded that increasing overall gambling, and specific gambling types during the COVID-19 crisis, is clearly associated with having a higher degree of gambling problems. Likewise, although a majority did not report an altered gambling behavior from the substantial changes to the world of sports, those who did report such an increase presented very high rates of gambling problems. In response to the COVID-19 crisis, those tending to increase gambling may have particular treatment needs, and given the potential vulnerability in that group, there is reason to take action in order to prevent crisis-related increases in gambling. The link between alcohol and gambling is particularly important to address and to prevent during the crisis.

## Figures and Tables

**Table 1:** Sample characteristics, all included individuals (N = 2016).

Sample Characteristics	n (%)
Gender	
Female	992 (49)
Male	1022 (51)
Prefer not to say	2 (0)
Age	
18–24 years	137 (7)
25–29 years	172 (9)
30–39 years	360 (18)
40–49 years	403 (20)
50–64 years	522 (26)
65 years and older	422 (21)
Living conditions	
With partner and children	527 (26)
With partner, no children	743 (37)
Without partner, with children	99 (5)
Without partner or children	550 (27)
Live with my parents	97 (5)
Employment/occupation	
Employed	1191 (59)
Job-seeking	82 (4)
Retired	465 (23)
Short-term unemployment	48 (2)
On sick leave	40 (2)
Studying	145 (7)
Other	45 (2)
Monthly income (SEK) ^1^	
Less than 10,000	160 (8)
10,000–15,000	204 (10)
15,000–20,000	187 (9)
20,000–25,000	228 (11)
25,000–30,000	290 (14)
30,000–35,000	300 (15)
35,000–40,000	235 (12)
40,000–45,000	131 (6)
45,000–50,000	92 (5)
Above 50,000	189 (9)
Past-year gambling, any time	
Online casino	205 (10)
Land-based casino	96 (5)
Horse betting online	374 (19)
Horse betting, land-based	280 (14)
Sports betting, online	390 (19)
Sports betting, land-based	260 (13)
Online poker	101 (5)
Electronic gambling machines, land-based	105 (5)
Online bingo	174 (9)
Monthly gambling losses, past month (SEK) ^1^	
0–49	1293 (64)
50–100	165 (8)
100–200	211 (10)
200–400	166 (8)
400–600	83 (4)
600–1000	165 (8)
1000–2000	31 (2)
above 2000	0 (1)
Monthly gambling losses, past month (SEK) ^1^	
0–49	1262 (63)
50–100	201 (10)
100–200	211 (10)
200–400	167 (8)
400–600	95 (5)
600–1000	43 (2)
1000–2000	26 (1)
above 2000	11 (1)
Kessler-6, total score ^2^	4 (1–8)

^1^ Local currency, Swedish krona (SEK). One SEK corresponds to around 0.11 Euros. ^2^ One or several of the six items were missing for 54 individuals (as a ‘prefer not to say’ option was possible for each of these items), not calculated in the median and inter-quartile range (IQR) values.

**Table 2:** Comparison of individuals reporting increased gambling, compared to all those reporting either unchanged gambling, decreased gambling or no gambling (full data, N = 2016), and compared to those reporting unchanged or decreased gambling (non-gamblers excluded, total N = 1246). Comparisons made with chi-square analysis for categorical data, and Mann-Whitney U test for continuous data.

Characteristics	Gambles More, n (%), (n = 74)	Does Not Gamble More, Full Sample, n (%), (n = 1942)	*p* Value, Gamble More vs. Not in Full Sample	Does Not Gamble More, Sub-Sample Excluding Non-Gamblers, n (%), (n = 1172)	*p* Value, Gamble More vs. Not in Sub-Sample
Male gender	43 (58)	979 (50)	0.19	692 (59)	0.87
Gambling severity					
no risk	17 (23)	1680 (87)	<0.001 ^1^	930 (79)	<0.001 ^1^
low risk	17 (23)	128 (7)		120 (10)	
moderate risk	15 (20)	61 (3)		56 (5)	
problem	25 (34)	73 (4)		66 (6)	
Age group (years)					
18–24	14 (19)	123 (6)	<0.001 ^1^	65 (6)	<0.001 ^1^
25–29	13 (18)	159 (8)		87 (7)	
30–39	16 (22)	344 (18)		196 (17)	
40–49	13 (18)	390 (20)		267 (23)	
50–64	9 (12)	513 (26)		346 (30)	
65 and above	9 (12)	413 (21)		211 (18)	
More time at home	66 (89)	1525 (79)	0.03	895 (76)	0.01
Irregular occupation (job-seeking, short-term unemployed, sick-leave)	10 (14)	160 (8)	0.11	97 (8)	0.12
Monthly income (SEK)					
Less than 10,000	6 (8)	154 (8)	0.79 ^1^	84 (7)	0.55 ^1^
10,000–15,000	10 (14)	194 (10)		99 (8)	
15,000–20,000	6 (8)	181 (9)		106 (9)	
20,000–25,000	10 (14)	218 (11)		139 (12)	
25,000–30,000	8 (11)	282 (15)		174 (15)	
30,000–35,000	9 (12)	291 (15)		186 (16)	
35,000–40,000	7 (9)	228 (12)		140 (12)	
40,000–45,000	7 (9)	124 (6)		82 (7)	
45,000–50,000	6 (8)	86 (4)		54 (5)	
Above 50,000	5 (7)	184 (9)		108 (9)	
Living alone, without children	20 (27)	530 (27)	0.96	309 (26)	0.9
Higher alcohol consumption	22 (30)	139 (7)	<0.001	85 (7)	<0.001
Self-exclusion from gambling, ever	21 (28)	49 (3)	<0.001	43 (4)	<0.001
Past-year gambling					
Online casino	35 (47)	170 (9)	<0.001	164 (14)	<0.001
Land-based casino	12 (16)	84 (4)	<0.001	72 (6)	<0.001
Online sports betting	39 (53)	351 (18)	<0.001	338 (29)	<0.001
Land-based sports betting	24 (32)	236 (12)	<0.001	217 (19)	<0.01
Online horse betting	36 (49)	338 (17)	<0.001	316 (27)	<0.001
Land-based horse betting	26 (35)	254 (13)	<0.001	209 (18)	<0.001
Online poker	21 (28)	80 (4)	<0.001	73 (6)	<0.001
Land-based electronic gambling machines	11 (15)	94 (5)	<0.001	81 (7)	0.01
Online bingo	31 (42)	143 (7)	<0.001	130 (11)	<0.001
Kessler-6 score	9 (6–14.5) ^2^	3 (1–8) ^3^	<0.001	4 (1–8) ^5^	<0.001
Kessler, moderate psychological distress ^4^	57 (77)	818 (43)	<0.001	509 (44)	<0.001

^1^ Chi-square, linear-by-linear ^2^ Score missing for one individual ^3^ Score missing for 53 individuals ^4^ Missing for 30 individuals ^5^ Score missing for 26 individuals.

**Table 3:** Logistic regression analyses examining correlates of reporting an increase in overall gambling, in all individuals with full data for all included variables (n = 1986), and for the sub-group including respondents who report no gambling neither during the COVID-19 crisis nor prior to it (n = 1233). Binary, non-stepwise regression analyses including all variables associated with gambling increase in bivariate analyses.

Potential Correlates	All Individuals (n = 1986). Odds Ratio (OR).	Odds Ratio 95-Percent Confidence Interval)	Sub-Sample, All but Non-Gamblers (N = 1233). OR.	Odds Ratio 95-Percent Confidence Interval
Problem severity	2.66	2.06–3.42	2.15	1.66–2.80
Older age group	1.02	0.84–1.24	0.97	0.80–1.19
Psychological distress	1.55	0.80–3.03	1.55	0.80–3.01
Self-exclusion	1.57	0.73–3.37	1.56	0.72–3.35
More time at home	1.62	0.74–3.56	1.75	0.80–3.84
Increased alcohol consumption	2.68	1.44–4.99	2.70	1.44–5.05

**Table 4:** Changes in specific gambling types. Calculated for the total of individuals excluding individuals reporting that they don’t engage in this particular gambling type, neither during COVID-19 nor prior to that. Number and percentages reporting an increase. Ratio of individuals reporting an increase vs a decrease for that gambling type.

Gambling Types	Total Number Excluding Non-Gamblers	Proportion Reporting an Increase, n (%)	Ratio Numbers Reporting Increase/Decrease
Online casino	295	36 (12)	0.62
Online sports betting	491	27 (5)	0.11
Land-based sports betting	546	25 (5)	0.12
Online horse gambling	555	75 (14)	0.76
Land-based horse gambling	438	28 (6)	0.19
Online lotteries	741	66 (9)	0.73
Land-based lotteries	1412	47 (3)	0.20
Land-based electronic gambling machines	335	21 (6)	0.26
